# Internationalconference honors pioneers in quantitative microbial physiology and ecology

**DOI:** 10.1093/nsr/nwaf316

**Published:** 2025-10-03

**Authors:** Qun Lu, Xuefei Li

**Affiliations:** Shenzhen Institutes of Advanced Technology, Chinese Academy of Sciences; Shenzhen Institutes of Advanced Technology, Chinese Academy of Sciences

## Abstract

The first Quantitative Microbial Physiology and Ecology (QMPE) Conference and a special ceremony for the 2nd Charles E. Helmstetter Prize was held in Shenzhen, China.

**Figure 1:**
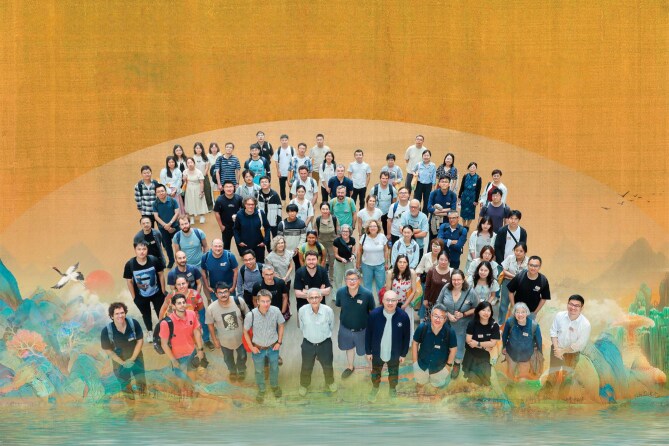


As an interdisciplinary frontier, microbial physiology and ecology systematically uncovers the quantitative principles of individual physiological responses and population-level ecological interactions by integrating single-cell analytics, multi-omics, and mathematical modeling. This provides innovative theoretical support for areas such as environmental remediation, healthcare, and synthetic biomanufacturing.

On 12–16 May 2025, the first Quantitative Microbial Physiology and Ecology (QMPE) Conference was held in Shenzhen, China. This event was hosted by the Shenzhen Institutes of Advanced Technology (SIAT), Chinese Academy of Sciences (CAS). It brought together researchers from 35 institutions from 10 countries, including China, the United States, the United Kingdom, France, the Netherlands, Switzerland, Italy, Israel, Japan, and India.

The conference featured four key topics: spatio-temporal coordination of bacterial DNA replication, mass growth and cell division; gene regulatory networks and physical processes underlying microbial physiology; microbial response and adaptation in changing environments; and ecology and evolution of microbial communities.

Additionally, a special ceremony for the 2nd Charles E. Helmstetter Prize, was held on the night of 14 May to make its debut announcement in China.

Charles E. Helmstetter, an Emeritus Professor at Florida Institute of Technology, is widely regarded as the pioneer who established the experimental and theoretical foundations of the bacterial cell cycle. His seminal work (1960s–late 20th century) shaped modern bacterial cell physiology research and set an enduring benchmark for scientific excellence. The Charles E. Helmstetter Prize award was established in 2022 through the collaborative efforts of Vic Norris (University of Rouen, France), Arieh Zaritsky (Ben-Gurion University of the Negev, Israel), and other specialists in the field. It aims to recognize and celebrate groundbreaking research that furthers our knowledge of the bacterial cell cycle.

During the prize ceremony, Arieh Zaritsky said that this award stands as a lasting tribute to Helmstetter's revolutionary contributions to the study of the bacterial cell cycle; and Vic Norris shared that one of the Prize's values is to help unite the field, which has become increasingly interdisciplinary.

The Prize Committee shares its membership with the QMPE organizing committee, including: Ariel Amir (Weismann Institute of Science), Marco Cosentino Lagomarsino (IFOM), Chenli Liu (SIAT, CAS), Wenying Shou (University College London), Jade Wang (University of Wisconsin-Madison), Jie Xiao (Johns Hopkins University). Among them, Liu chaired the Prize Committee, with Johan Elf (Uppsala University) serving as an additional member.

This year's Helmstetter Prize honors two leading scientists for their groundbreaking contributions to the field of microbial physiology: the Lifetime Achievement Award goes to Prof. Nancy Kleckner (Harvard University, US), and the Innovation Breakthrough Award goes to Prof. Nathalie Balaban (Hebrew University of Jerusalem, Israel).

‘The two awardees have advanced microbial physiology through interdisciplinary innovation, embodying Prof. Helmstetter's vision that “fundamental research drives major breakthroughs.” The award not only recognizes outstanding scientific achievements but also honors and continues the legacy of pioneering scientists in the field,’ said Liu.

Prof. Nancy Kleckner is a member of the US National Academy of Sciences and is renowned for her pioneering research on dynamic chromosomal processes in *E. coli* and eukaryotic cells. She discovered SeqA, a protein crucial in DNA replication initiation, significantly advancing the field of bacterial cell cycle research and broadening our understanding of chromosomal behavior across various organisms.

Prof. Nathalie Balaban is a renowned biophysicist and systems biologist. Using innovative biophysical approaches, she has uncovered bacterial response mechanisms under antibiotic stress, providing new insights into the dynamics of stress adaptation and offering potential strategies to address the global challenge of antimicrobial resistance.

The ceremony featured a special online discussion with Prof. Helmstetter, who congratulated the two awardees of the 2nd Prize and emphasized that the prize aims to ensure continued support for outstanding work in the field. Additionally, he expressed his deep appreciation for Arieh Zaritsky, who organized a remarkable series of 16 EMBO workshops on bacterial physiology over 45 years, culminating in 2022. He noted that ‘it is time for the youth to take over, and I am pleased they have,’ expressing his hope that ‘the series of meetings can continue for the next 45-plus years.’

The first QMPE conference provided a unique and intimate platform for researchers in the fields of microbial physiology and ecology to exchange insights, foster collaborations, and explore future research directions. The 2nd QMPE will be held in 2027, with the aim of continuously strengthening the microbial physiology community.

This conference was supported by the American Society for Microbiology (ASM), Agilent Technologies Co., Ltd., Bruker Corporation, FutuRay Technology Development Co., Ltd. of Shenzhen, the National Industrial Innovation Center for Bio-manufacturing, and the Shenzhen Synthetic Biology Association.

Qun Lu is a Project Manager in the Office of International Cooperation at SIAT, CAS. Xuefei Li is Professor at SIAT, CAS.

